# Isotype-selective roles of hepatic acetyl-CoA carboxylases in a mouse model of fatty liver disease

**DOI:** 10.1016/j.molmet.2025.102264

**Published:** 2025-10-04

**Authors:** Martina Beretta, Calum S. Vancuylenburg, Riya Shrestha, Ellen M. Olzomer, Brenna Osborne, Mingyan Zhou, Suri Zhang, Adam Hargreaves, Frances L. Byrne, Kyle L. Hoehn

**Affiliations:** 1 School of Biotechnology and Biomolecular Sciences, University of New South Wales, Kensington, NSW 2033, Australia; 2 PathCelerate Ltd, Goostrey, UK

**Keywords:** Liver, Obesity, Mitochondria, Amylin, MASH, MASLD

## Abstract

**Objectives:**

Acetyl-CoA carboxylase enzymes ACC1 and ACC2 promote liver fat storage. Accordingly, ACC inhibition represents a strategy to reverse fatty liver disease and related disorders. Human and rodent studies show that targeting both ACC isotypes can reverse some fatty liver phenotypes, but also result in unwanted metabolic phenotypes including hypertriglyceridemia. The objective of this study was to determine whether liver-selective genetic inhibition of ACC1 or ACC2 individually can reverse fatty liver disease phenotypes without adverse metabolic phenotypes in a mouse model of fatty liver disease.

**Methods:**

Four genotypes of male C57BL/6J mice floxed for ACC1, ACC2, both ACC alleles, or no ACC alleles were fed an Amylin diet for 28 weeks to induce fatty liver disease. After 20 weeks of Amylin feeding, ACC genes were deleted in the liver by adeno-associated virus 8 (AAV8)-mediated Cre recombinase expression. Mice were metabolically phenotyped and liver disease was assessed by histopathology.

**Results:**

Dual inhibition of ACC enzymes was necessary to achieve significant reversal of fatty liver disease and fibrosis; however, it also caused hypertriglyceridemia, weight gain, and glucose intolerance. ACC1 inhibition alone resulted in partial reversal of fatty liver disease phenotypes but drove all undesired metabolic phenotypes. In contrast, ACC2 inhibition alone had minimal effect on fatty liver, fibrosis, or metabolic phenotypes.

**Conclusions:**

Our results indicate that complete inhibition of liver ACC activity is required to resolve fatty liver disease and fibrosis, with ACC1 inhibition being the dominant driver of unwanted metabolic dysregulation. Accordingly, selective inhibition of ACC2 with partial inhibition of ACC1 may represent a refined future approach to reverse fatty liver disease phenotypes while minimizing metabolic dysregulation.

## Highlights

• Hepatic ACC1 and ACC2 deletion in adult mice reverses steatosis and fibrosis.
• Hepatic ACC1 and ACC2 deletion triggers several undesired metabolic phenotypes.
• Hepatic ACC1-specific deletion drives metabolic dysregulation with little efficacy.
• Hepatic ACC2-specific deletion has no metabolic dysregulation but no efficacy.
• Total ACC deletion effectively reverses MASH but ACC1 loss impairs metabolism.

## Graphical abstract

**Figure:**
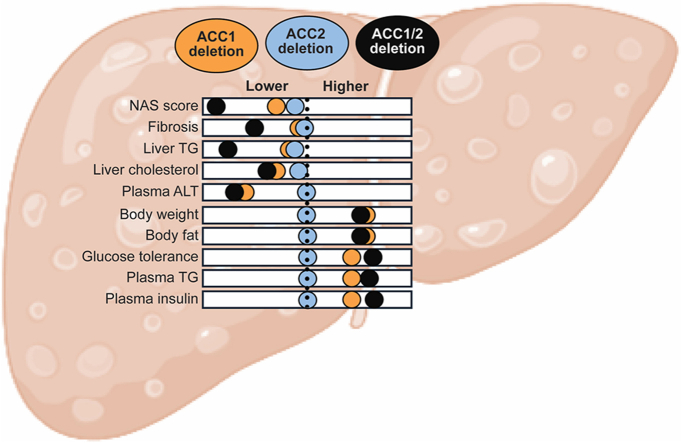

## 1 Introduction

Metabolic dysfunction-associated steatotic liver disease (MASLD), formerly known as non-alcoholic fatty liver disease (NAFLD), is emerging as a major health challenge worldwide with a prevalence of 30% among adults [1,2]. In addition, the rates of obesity and MASLD are increasing steadily among adolescents [3], leading to an earlier onset and longer duration of MASLD and associated complications in the lifetime of individuals. MASLD encompasses a spectrum of liver disorders starting from steatosis, which is primarily characterized by excessive fat accumulation in hepatocytes (>5%), with at least one of five cardiometabolic risk factors. MASLD can progress to metabolic dysfunction–associated steatohepatitis (MASH) where inflammation and cellular injury (hepatocyte ballooning) are present, with or without fibrosis [4]. Liver fibrosis is the primary determinant of mortality in patients with MASH [5] due to increased liver-related complications including higher risk of hepatocellular carcinoma [6,7].

Lifestyle changes such as diet and exercise remain the cornerstones of MASH treatment. Clinical studies have shown that reducing body weight by ≥ 10% can promote MASH resolution and fibrosis regression [8,9], making this a critical component of therapy. However, sustained weight loss due to lifestyle change alone is challenging and pharmacological treatments for weight loss including GLP-1R agonists are increasingly being used for both obesity and MASH. For example, the GLP-1R agonist semaglutide received accelerated FDA approval in August 2025 for the treatment of MASH with moderate fibrosis (F2–F3), but it is to be used in conjunction with lifestyle interventions of calorie restriction and exercise. Semaglutide is the second FDA-approved MASH treatment following accelerated approval of the thyroid hormone receptor-β agonist resmetirom (Rezdiffra) in March 2024 for the treatment of MASH with moderate fibrosis (F2–F3) [10,11].

Despite these two new treatments for MASH, many patients remain refractory to treatment. For example, 72 weeks of semaglutide treatment (ESSENCE trial) at 2.4 mg weekly resulted in 63% MASH resolution vs 34% resolution for placebo, with 37% of patients showing at least one stage improvement in fibrosis without worsening MASH compared to 22% of placebo patients. With resmetirom treatment at 80–100 mg daily for 52 weeks (MAESTRO-NASH trial), only ∼26–30% of patients responded with MASH resolution and no worsening of fibrosis vs 9.7% for placebo. Further, 24–26% of patients showed at least one stage improvement in fibrosis without worsening MASH compared to 10–14% of patients receiving placebo. Accordingly, current treatments have a placebo-adjusted efficacy for MASH resolution of less than 29% and fibrosis reversal of less than 15%. These data highlight the need for more effective treatments to tackle this multifactorial and heterogeneous disease.

Strong evidence indicates that increased *de novo* lipogenesis (DNL) contributes to hepatic fat accumulation in patients with MASLD [12,13]. DNL is the biochemical pathway that synthesizes fatty acids from carbohydrate and amino acid precursors via acetyl-coenzyme A (CoA) substrate. Acetyl-CoA carboxylases (ACC) catalyse the first and rate-limiting step of the DNL pathway that involves carboxylation of acetyl-CoA to produce malonyl-CoA [14]. In mammals, two ACC isotypes exist including ACC1 and ACC2. ACC1 is a cytosolic enzyme predominantly expressed in lipogenic tissues (e.g. liver and adipose tissue), and ACC2 is localized to the outer mitochondrial membrane in oxidative tissues (e.g. liver, skeletal muscle, and heart) [15]. Studies using ACC knockout (KO) mouse models and antisense oligonucleotide (ASO)-treated rats demonstrated that malonyl-CoA produced by ACC1 is primarily consumed to drive DNL while malonyl-CoA produced by ACC2 mainly acts to repress fat oxidation by inhibiting fat entry into mitochondria via carnitine palmitoyltransferase 1 (CPT1) [16]. However, there is evidence that each isotype can partially compensate for the loss of the other in liver [17].

ACC inhibitors have emerged as an attractive approach to treat MASLD because they simultaneously decrease DNL and promote fatty acid oxidation, with an overall effect of decreased hepatic steatosis [15]. Moreover, by effectively resolving MASH and improving fibrosis, these therapies appear to meet the highest endpoint FDA guidelines for MASH drug development among existing candidates [18]. However, studies have shown that liver targeting of ACC-based therapies is necessary to avoid unwanted declines in platelet counts [19]. Liver targeted ACC inhibitors, including firsocostat (also known as GS-0976 and ND-630) from Gilead, clesacostat (also known as PF-05221304) from Pfizer, and MK-4074 from MSD all showed robust capacity to lower hepatic fatty acid accumulation and fibrosis in clinical trials [[20], [21], [22], [23]]. Nonetheless, 8–18% individuals treated with these drugs developed significant hypertriglyceridemia, resulting in withdrawal of the participants from clinical trials. Elevated serum triglycerides increase the risk of cardiovascular disease and pancreatitis, potentially compromising the clinical benefits of ACC inhibitors. Strategies that effectively mitigate ACC inhibitor-induced hypertriglyceridemia will therefore be critical for the development of this class of drugs unless an ACC isotype-selective drug strategy can result in the reversal of MASH without unwanted adverse phenotypes.

Although informative, previous studies with whole-body or liver-targeted ACC KO mice have produced contrasting results and have not been ideal for predicting the therapeutic outcomes of ACC inhibition, partly because the loss of enzyme activity occurs from birth and persists throughout the mouse lifespan (i.e. a MASH prevention model rather than a reversal model) and/or the chosen model does not accurately reflect the complex pathophysiology of human MASH. Additionally, the distinct roles of hepatic ACC isotypes in the pathophysiology of MASH and the regulation of serum triglyceride have yet to be elucidated. In the present study, we assessed for the first time the impact of both liver-targeted and isotype-selective ACC inhibition in a diet-induced mouse model of MASH. By taking advantage of conditional ACC1 and ACC2 floxed mice, we achieved liver-specific and timed ACC deletion after the onset of MASH. We found that complete inhibition of liver ACC activity is required to reverse MASH and liver fibrosis, with the caveat that it caused i) increased serum triglycerides, consistent with previous studies on dual liver-targeted ACC inhibitors [[20], [21], [22], [23]], ii) increased adiposity, and iii) glucose intolerance. Moreover, our results suggest that while hepatic ACC1 deletion is not enough to resolve MASH or liver fibrosis, it is sufficient to drive hypertriglyceridemia, body weight gain and glucose intolerance.

## 2 Materials and methods

### 2.1 Mouse breeding and husbandry

Mouse experiments for this study were approved by the UNSW Animal Care and Ethics Committee (approvals 20/43A and 23/56A). Floxed ACC1 and ACC2 mice were generated as previously described [24,25]. Briefly, floxed ACC1 and ACC2 genes were engineered in C57BL/6 embryonic stem cells to generate all mouse crosses on a pure C57BL/6 background. Founder mice were also crossed with C57BL/6 mice expressing FLPe to delete embryonic stem cell selection cassettes. Mice were bred and maintained at ABR (Moss Vale, NSW, Australia) and delivered to the Wallace Wurth animal facility at UNSW at 5 weeks of age to acclimatize to laboratory conditions for one week prior to study start. Four different genotypes were analyzed in this study: ACC1^+/+^/ACC2^+/+^; ACC1^fl/fl^/ACC2^+/+^; ACC1^+/+^/ACC2^fl/fl^, and ACC1^fl/fl^/ACC2^fl/fl^. Animals were housed at 22 °C in a light–dark cycle of 12 h, monitored weekly and had ad libitum access to water and diet. Body composition measurements were assessed weekly for 10 weeks prior to the end of the study using the EchoMRI-900 (EchoMRI LLC, Houston, TX, USA) at the Biological Resource Imaging Laboratory (UNSW Sydney, Australia). At the end of the study, mice were anesthetized with isoflurane and terminal bloods were collected via cardiac puncture in EDTA-capillary tubes (Sarstedt, 15.1671.100).

### 2.2 Mouse diets

From 6 weeks of age, male mice of all genotypes were fed Amylin liver NASH diet for 28 weeks in total. As a reference for lean healthy physiology, we also included a group of fifteen mice fed normal chow diet (catalog SF00-100, Gordons Specialty Feeds, New South Wales, Australia). The chow group consisted of male age-matched littermate controls of mixed flox genotypes with no AAV treatment. The Amylin diet was prepared in-house using a recipe adapted from Research Diets #D09100301: caloric content was 46% fat kcal (of which 15% was trans-fat by weight), 36% sugar (of which 22% was fructose and 10% was sucrose by weight) and 18% protein. Diet ingredients were purchased from local suppliers. For diet preparation, we used: primex shortening (Dyets, 403760), fructose (Melbourne Food Depot, FRUCT10K), cholesterol (Sigma, C8503, 2% by weight), sucrose (JL Stewart, GRAD25B), corn starch (JL Stewart, CFLR25W), wheat bran (JL Stewart, BRAN10UF), casein (Cottee Group, NA), choline bitartrate (Sigma, C1629), lard (JL Stewart, LARD15), soybean oil (Masterol Foods, 165194538), trace minerals (MP Biomedicals, 0296026401), AIN-93M mineral mix (MP Biomedicals, 0296040102) and AIN-93-VX vitamin mix (MP Biomedicals, 0296040201).

### 2.3 AAV8 virus treatments

At 8 weeks prior to the end of the study, Amylin diet-fed mice were injected intravenously with a recombinant adeno-associated virus 8 (AAV8) expressing Cre recombinase via a hepatocyte-specific thyroxine-binding globulin (TBG) promoter (AAV8-TBG-Cre) or AAV8-TBG-eGFP as virus negative control. AAV8 viral vectors expressing Cre-recombinase or eGFP were purchased from Penn Vector Core (AV8.TBG.PI.Cre.rBG, titre 9.15eˆ13 GC/mL, Lot # CS2188L; AAV8.TBG.PI.eGFP.WPRE.bGH, titre 1.24eˆ14 GC/mL, Lot # CS2189L, University of Pennsylvania, Philadelphia, USA). The AAV8 vectors were diluted on the day of the procedure in saline to make up 10eˆ10 particles in a 5 μL/g of body weight final volume. Chow-fed animals were injected with saline as controls. At the end of the study, ACC enzyme deletion was confirmed for all animals by western blotting.

### 2.4 Western blotting

Frozen liver tissues were powdered and protein lysates prepared by homogenizing tissues in NP-40 buffer (50 mM Tris HCl pH 7.5, 150 mM NaCl, 10% glycerol and 1% NP-40) + protease inhibitors (Sigma, #11697498001). Fifty μg of protein/sample were denatured in Laemmli buffer and loaded onto 6% acrylamide gels before SDS-PAGE gel electrophoresis and protein transfer to a nitrocellulose membrane. The membrane was blocked with 1% bovine serum albumin (BSA) powder in TBS (20 mM Tris Base and 150 mM NaCl) and incubated with rabbit polyclonal ACC antibody (3662, Cell Signaling, USA) in 0.1% BSA in TBS-T (20 mM Tris Base, 150 mM NaCl and 0.1% (w/v) Tween 20) at 1:1000 dilution. Mouse anti-vinculin (V9131, Sigma–Aldrich, USA) was used as a loading control. Blots were then probed with donkey anti-rabbit IgG H&L (Alexa Fluor® 680 preabsorbed, ab186692, Abcam, USA) and donkey anti-mouse IgG H&L (Alexa Fluor® 790 preabsorbed, ab186699, Abcam, USA) in TBS-T at 1:5,000 dilution. Bands were visualized and quantified by fluorescence imaging using an Odyssey® DLx Imaging System (LI-COR Bioscience, USA).

### 2.5 Glucose tolerance tests (GTT) and plasma samples collection

Glucose tolerance tests (GTT) were performed the week before AAV8 injections (pre-treatment) and two weeks before the end of the study (post-treatment). Mice were fasted for 5 h during the day, and blood collection was performed from a tail nick into heparin-coated tubes. For GTTs, 2 g of glucose per kilogram of lean mass was injected intraperitoneally in the form of a 33.3% glucose solution prepared in sterile saline. Blood glucose levels were measured at baseline and 15, 30, 45, 60, 90 and 120 min after glucose administration using an Accu-Chek® Performa II blood glucose meter (Roche, Germany). Total area under the curve (AUC) was calculated using the trapezoidal method.

Random-fed (RF) and 6-hour fasted blood samples were collected in heparin-coated tubes (Sarstedt, 16.443), and blood glucose evaluated 2 weeks before AAV8 injections as well as 4 weeks before the end of the study using an Accu-Chek® Performa glucometer. Samples were centrifuged at 2,000×*g*, and plasma was stored at −80 °C for later analyses.

### 2.6 Liver histology and NAFLD score assessment

A section of the liver left lateral lobe was collected and fixed in 10% neutral buffered formalin (NBF) overnight for histological assessment. Formalin-fixed liver samples were paraffin-embedded, sectioned at 4 μm thickness and stained with hematoxylin-eosin or picrosirius red staining by the Katharina Gaus Light Microscopy Facility at UNSW (NSW, Australia). The NAFLD activity score (NAS) system was applied to all samples for scoring of steatosis, lobular inflammation and hepatocyte ballooning as outlined by Kleiner et al. [26]. Collagen deposition (fibrosis stage) was graded based on picrosirius red staining evaluation. Histological assessments were performed by the independent contract research organization PathCelerate (Goostrey, Cheshire, UK) and all assessments were performed by a pathologist blind to treatment.

### 2.7 Lipid and biochemical analysis

Liver tissues were snap-frozen in liquid nitrogen immediately after collection and stored at −80 °C until analysis. Lipids were extracted from powdered livers using a modified version of Folch et al., 1957 method [27] as previously described [28]. Briefly, liver lipids were extracted from approximately 25 mg of tissue with 2:1 chloroform-methanol v/v, following by two washing steps of the lipid-rich lower phase in saline. Lipid extracts were dried under a steady stream of nitrogen in a TurboVap Evaporator (Biotage) and re-suspended in 0.4 mL 95% ethanol. Following 10 min heating at 37 °C, lipid extracts along with plasma samples were used to measure triglycerides and cholesterol content via colorimetric assay following manufacturer's instructions (Pointe Scientific, T7532; Thermo Scientific, TR13421). For liver tissue samples, results obtained were normalized to initial grams of powder extracted.

Plasma insulin concentration was measured in 6-hour fasted samples collected over the course of the study using Crystal Chem Ultra-Sensitive Mouse Insulin ELISA kit (Catalog number 90080) following manufacturer's instructions. The HOMA-IR insulin resistance index was calculated as follows: Fasting glucose (mg/dL) × Fasting insulin (mU/mL)/450; as has been validated in rodents [29,30]. Alanine transaminase (ALT) plasma levels were measured using an ALT activity assay (MAK052, Sigma) on random fed plasma samples collected during the study. The assay was performed as a fluorometric test using 2.5 μL (1:8 dilution in ALT assay buffer) of heparinized plasma samples. Plasma free fatty acid (FFA) concentration was measured in 6-hour fasted samples collected over the course of the study using the EnzyChrom free fatty acid assay kit (EFFA-100, BioAssay Systems, fluorometric mode).

### 2.8 Quantitative real-time PCR (qRT-PCR)

RNA extraction was performed on 50–100 mg powdered liver samples using a TRI reagent according to the manufacturer's protocol (Sigma T9424, Castle Hill, NSW, Australia). cDNA synthesis was performed using 2000 ng RNA, a DNAse I kit (Sigma AMPD1) and a High-Capacity cDNA Reverse Transcription Kit (Applied Biosystems, # 4368814), according to the manufacturer's protocols. PCR products were amplified in triplicates using 384-well plates in the following (5 μL) reaction: 0.33 μL of each primer (from 100uM stocks), 2.5 μL iTaq Universal SYBR Green Supermix (Bio-Rad, # 1725124), 1.17 μL nuclease-free water, and 0.67 μL cDNA. An Applied Biosystems ViiA7 Real-Time PCR System (Thermofisher, Waltham, MA, USA) was used and PCR cycling conditions were as follows: 95 °C (20 s), 40 cycles (95 °C (1 s) and 60 °C (20 s)) and melt curve stage (95 °C (15 s), 60 °C (1 min), and 95 °C (5 s)). qPCR was conducted using the primer sequences listed in Table 1; all primers were purchased from Sigma. Relative mRNA expression was calculated according to the ΔΔCt method. Relative values were normalized to 18S ribosomal RNA expression level as a housekeeping gene.

**Table 1:** Primer sequences of mouse genes used for qPCR.

Gene	Forward primer 5’ → 3′	Reverse primer 5’ → 3′	Amplicon (bp)
*Col1a1*	tccggtgtgactcgtgcagc	ccagctgaccttcctgcgcc	211
*Acta2*	gtcccagacatcagggagtaa	tcggatacttcagcgtcagga	102
*Loxl2*	attaaccccaactatgaagtgcc	ctgtctcctcactgaaggctc	130
*Mcam*	cccaaactggtgtgcgtctt	ggaaaatcagtatctgcctctcc	220
*Timp2*	cttggttccctggcgtactc	acctgatccgtccacaaacag	150
*Mmp2*	tggcagtgcaatacctgaac	ccgtacttgccatccttctc	670
*Acaca*	catcaccatcagcctggttaca	actgtgtacgctcttcggcat	283
*Acacb*	gtttgggcactgcttctcct	cacacaccaccccaagcat	236
*18S*	ttccagcacattttgcgagta	cccttaatggcagtgatggc	80

### 2.9 Statistical analyses

All data are presented as the mean ± standard deviation (SD). Statistical testing was performed using Prism (version 10.2.0; GraphPad software), where the threshold for significance was determined to be reached when p < 0.05 compared to WT controls fed Amylin diet. Normality distribution was tested using the Shapiro–Wilk test. For normally distributed data, differences between groups were examined using a one-way analysis of variance (ANOVA) with Dunnett's post hoc test for multiple comparisons. For non-parametric data, multiple groups were analyzed by Kruskal–Wallis test with Dunn's post-hoc correction. For multiple groups measured over time, differences between groups were examined by using two-way repeated measures ANOVA using Dunnett's post hoc test for multiple comparisons compared to the WT controls fed Amylin diet.

## 3 Results

### 3.1 Model validation

In this study, we evaluated the effects of selective and liver-targeted ACC1 and/or ACC2 inhibition in a mouse model of MASH induced by chronic Amylin diet feeding. The Amylin diet induces steatosis, inflammation, ballooning, and fibrosis [31] that resembles the pathophysiologic progression of human MASH in only 20–30 weeks [32,33].

**Figure 1:**
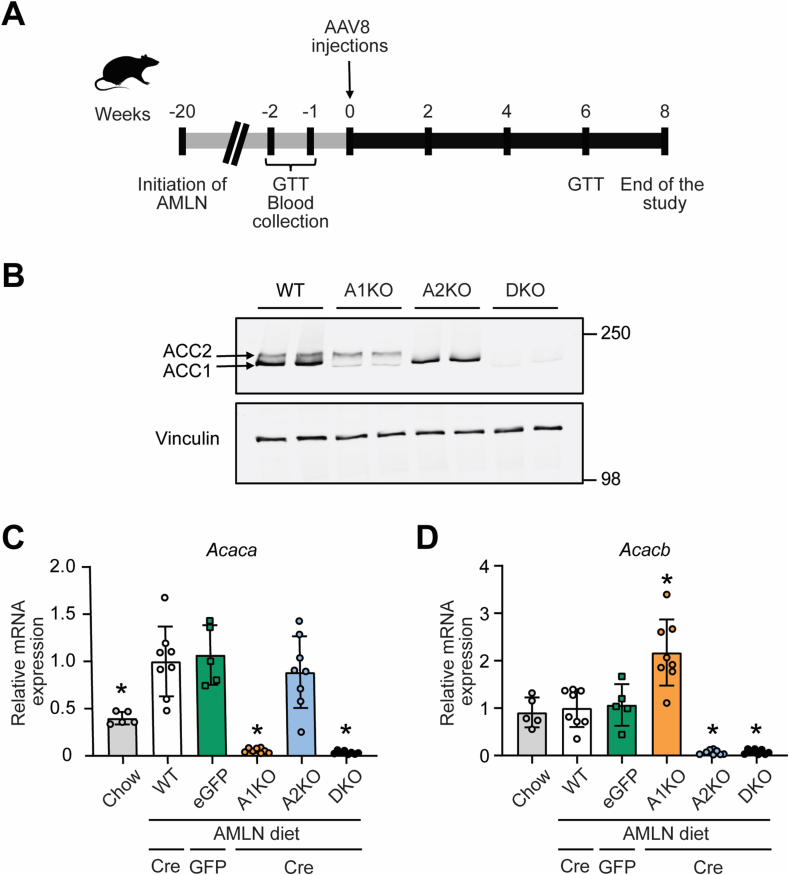
**Experimental design and confirmation of hepatocyte-specific ACC1 and ACC2 deletion.** (A) Schematic overview of the MASH animal study in male C57BL/6J mice. (B) Representative western blot images of ACC1, ACC2, and vinculin protein expression in mouse livers from wild-type (WT), ACC1 KO (A1KO), ACC2 KO (A2KO) and ACC1/2 KO (DKO) mice (all mice received AAV8-Cre virus). (C,D) Gene expression levels of (C) ACC1 and (D) ACC2. AAV8 virus treatments are indicated as Cre, GFP, or no virus. ∗indicates p < 0.05 compared to WT, determined by one-way ANOVA followed by Dunnett's multiple comparison post hoc test. Values are represented as mean ± SD, n = 5–8. Abbreviation: AMLN, Amylin diet.

Figure 1A illustrates the study timeline. From 6 weeks of age, floxed and control mice were fed Amylin diet for 28 weeks in total. At eight weeks prior to the end of the study, mice were administered AAV8-TBG-Cre virus to produce ACC1 knockout (A1KO), ACC2 knockout (A2KO), ACC1/ACC2 double knockout (DKO), or wild-type (WT) mice. A group of Amylin diet fed mice of mixed flox genotypes was injected with AAV8-TBG-eGFP as a control (eGFP), while a separate group of mice (mixed flox genotypes) were injected with saline and fed chow diet as a reference control for normal physiology.

Liver tissue from all mice treated with AAV8-Cre viruses were evaluated for ACC1 and ACC2 protein expression to validate the expected gene knockouts of ACC1, ACC2, both ACC isotypes or none (Fig. 1B). Separately, we showed that AAV8-Cre and AAV8-GFP treatments did not alter ACC enzyme expression compared to control mice without virus treatment (Fig. S1A). ACC mRNA levels were assessed by qPCR (Figure 1C,D), demonstrating deletions of ACC genes with a residual level of mRNA (∼3–8% vs WT) likely due to non-hepatocyte cells. Of note, liver-targeted deletion of ACC1 resulted in a 2-fold increase in ACC2 mRNA (Fig. 1D).

To determine the liver-selective expression of AAV8-TBG-Cre, we also evaluated ACC protein expression in quadriceps muscle and gonadal adipose tissues. Quadriceps muscle primarily expresses ACC2 and we observed no loss of muscle ACC2 expression in mice that received AAV8-TBG-Cre virus (Fig. S1B). Adipose tissue primarily expresses ACC1 and we observed no loss of adipose ACC1 expression in mice that received AAV8-TBG-Cre virus (Fig. S1C). These data demonstrate that AAV8-TBG-Cre virus selectively targets the liver without deleting ACC genes in other key metabolic tissues including muscle or fat.

We have previously published liver-specific double ACC1/ACC2 knockout mice where floxed ACC1 and ACC2 gene deletion in liver was driven by Albumin-Cre [24]. In that study, two markers of lipid metabolism and mitochondrial function were altered by liver ACC ablation including lower gene expression of the rate limiting enzyme in fatty acid oxidation CPT1a and increased activity of citrate synthase [24]. The mice in the present manuscript differ from the previous model by the use of AAV8-Cre virus to knockout floxed ACC genes in adult mice (in contrast to neonatal deletion by albumin-Cre) and herein we deleted each ACC isotype individually as well as together. Therefore, we assessed the functional effects of ACC gene deletion in all groups by assessing liver *Cpt1a* expression and citrate synthase activity. We found that DKO mice had decreased liver *Cpt1a* expression and increased citrate synthase activity; however, no phenotypes were observed in A1KO or A2KO mice (Fig. S2). These data demonstrate reproducibility of the fat metabolism and mitochondrial phenotypes across similar models and also show that both enzymes must be inhibited to mediate these phenotypes.

### 3.2 Hepatic ACC1 and ACC2 deletion improves liver markers of MASH

**Figure 2:**
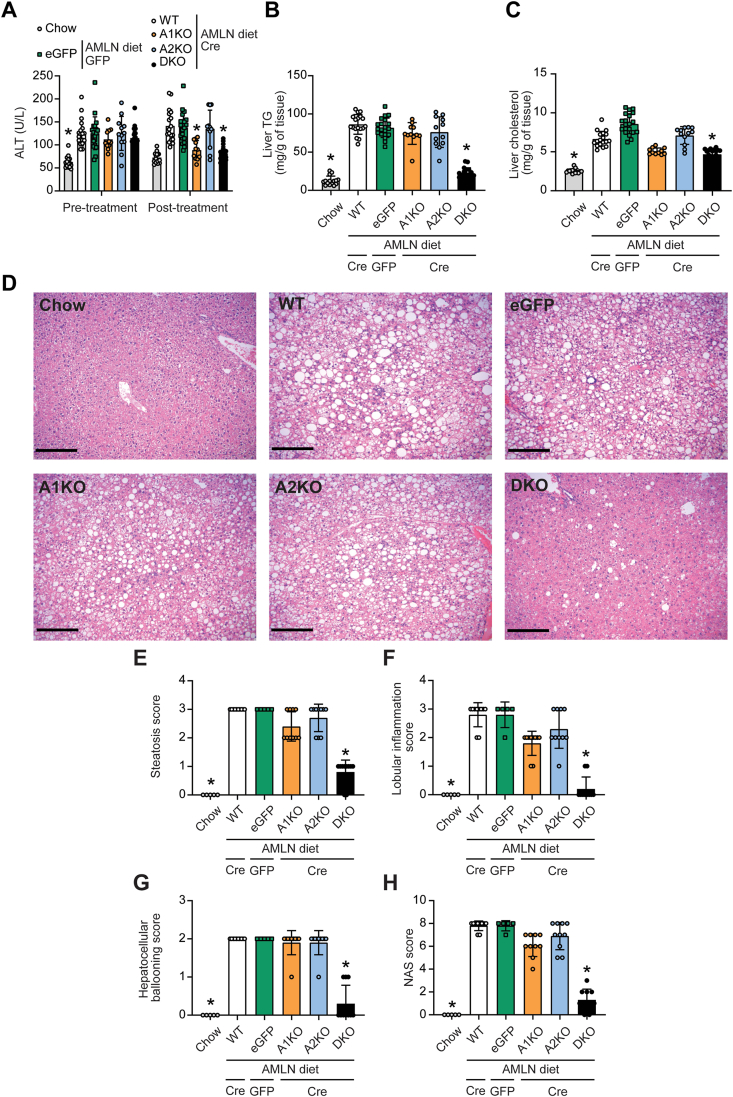
**Hepatic effects of ACC1 and ACC2 deletion in mice fed Amylin diet.** (A) Alanine transaminase (ALT) plasma levels measured pre- and post-AAV8 injections (pre-treatment at week −2 and post-treatment at week 4). ∗indicates p < 0.05 compared to WT, determined by two-way repeated measures ANOVA followed by Dunnett's multiple comparison post hoc test. Liver (B) triglyceride (TG) and (C) cholesterol levels measured at the end of the study. ∗indicates p < 0.05 compared to WT, determined by Kruskal–Wallis test followed by Dunn's multiple comparison post hoc test. (D) Representative images of liver H&E staining (scale bar is 250 μm). Histological grading of (E) liver steatosis, (F) lobular inflammation, (G) hepatocellular ballooning and (H) NAFLD Activity Score (NAS). ∗indicates p < 0.05 compared to WT, determined by Kruskal–Wallis test followed by Dunn's multiple comparison post hoc test. Values are represented as mean ± SD, n = 11–20. Abbreviation: AMLN, Amylin diet.

We next measured common markers associated with MASLD, including plasma alanine transaminase (ALT) levels and lipid content. ALT is a circulating marker of liver damage that is increased in all mice fed Amylin diet compared to chow-fed controls measured at the pre-treatment time point. Reassessment of ALT levels at 4 weeks post-AAV8 treatment showed that both ACC1 and dual ACC1/2 knockout significantly decreased ALT levels to near normal concentrations of chow-fed controls (Figure 2A). Liver TG levels were significantly normalized to levels comparable to chow-fed controls by dual ACC1/2 deletion, while A1KO and A2KO mice showed small non-significant trends towards TG reduction compared to WT controls (Fig. 2B). Additionally, liver cholesterol levels were significantly lower in DKO mice compared to WT controls while A1KO mice had an intermediate phenotype with a non-significant but trend for lower liver cholesterol (Fig. 2C). In contrast to liver, there were no statistically significant differences in TG or cholesterol in quadriceps muscle tissue across any genotypes fed Amylin diet (Fig. S3).

Liver histology (Fig. 2D) was scored for liver steatosis (Fig. 2E), lobular inflammation (Fig. 2F) and hepatocellular ballooning (Fig. 2G) using clinical criteria outlined by Kleiner et al. [26]. The sum of these three histological features was calculated for each sample as the NAFLD Activity Score (NAS) (Fig. 2H). Of all mice fed Amylin diet, the DKO mice had the best histopathology and lowest NAS score. Specifically, DKO mice showed a strong > 2-point decrease in liver steatosis, > 2-point decrease in lobular inflammation, and >1.5-point decrease in hepatocellular ballooning scores (Figure 2E–G) with an overall marked decrease in NAS score of > 6-points compared to WT and eGFP controls (Fig. 2H). In contrast, isotype-specific deletion of ACC1 or ACC2 did not statistically improve any NAS parameter although there was a non-statistical trend for lower inflammation and steatosis with ACC1 deletion.

### 3.3 Complete ACC deletion in the liver markedly improves liver fibrosis

**Figure 3:**
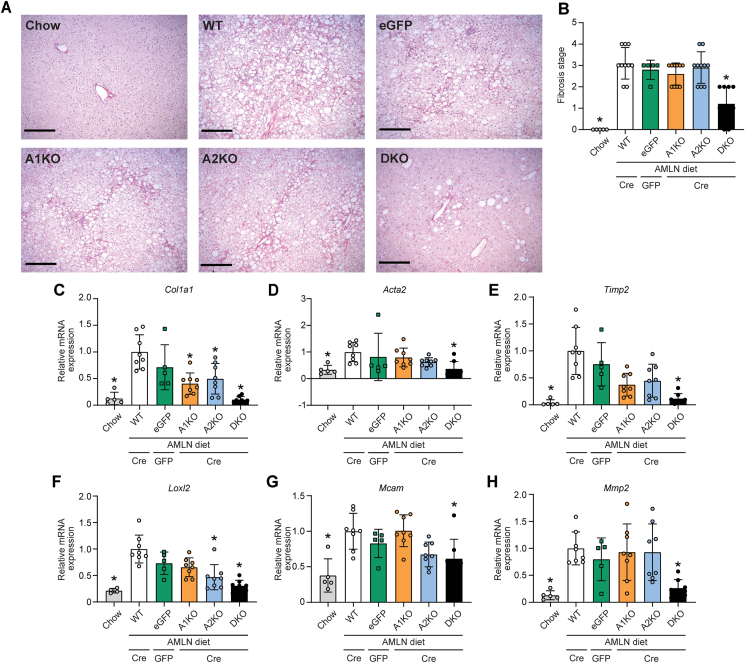
**Liver fibrosis in A1KO, A2KO and DKO mice.** (A) Representative images of liver picrosirius red staining (scale bar is 250 μm). (B) Histological grading of liver fibrosis stage. (C–H) Gene expressions of (C) collagen1 α1 (*Col1a1*), (D) alpha-smooth muscle actin (*Acta2*), (E) tissue inhibitor of metalloproteinases 2 (*Timp2*), (F) lysyl oxidase-like homolog 2 (*Loxl2*), (G) melanoma cell adhesion molecule (*Mcam*), and (H) matrix metalloproteinase-2 (*Mmp2*). ∗indicates p < 0.05 compared to WT, determined by Kruskal–Wallis test followed by Dunn's multiple comparison post hoc test. Values are represented as mean ± SD, n = 11–20. Abbreviation: AMLN, Amylin diet.

We next examined the anti-fibrotic effects of hepatic ACC deletion by measuring hepatic collagen deposition (Figure 3A). As expected, chow-fed mice had no fibrosis while the WT and eGFP control liver tissues showed multi-focal areas of bridging fibrosis (fibrosis stage 3). The only test group to show significant improvements in fibrosis was the DKO group that mostly displayed periportal or centrilobular fibrosis (fibrosis stage 1) with an overall fibrosis grading that was significantly decreased as compared to WT and eGFP controls (Fig. 3B). In addition, DKO livers showed robust suppression of 6 gene expression markers of fibrosis including hepatic extracellular matrix (ECM) components and metalloproteinases (Figure 3C–H). Histological analyses did not show any improvement of liver fibrosis stage in A1KO and A2KO mice (Fig. 3B). However, gene expression data showed an intermediate phenotype for the isotype-selective KO mice whereby A1KO and A2KO livers had a significant decrease in collagen1 α1 mRNA levels (Fig. 3C), A2KO livers had lower lysyl oxidase-like homolog 2, and both genotypes had a general trend towards decreased gene expression of hepatic ECM organization genes such as alpha-smooth muscle actin and tissue inhibitor of metalloproteinases 2 (Figure 3D–H).

### 3.4 Hepatic ACC1 deletion triggers unwanted metabolic phenotypes in mice fed Amylin diet

**Figure 4:**
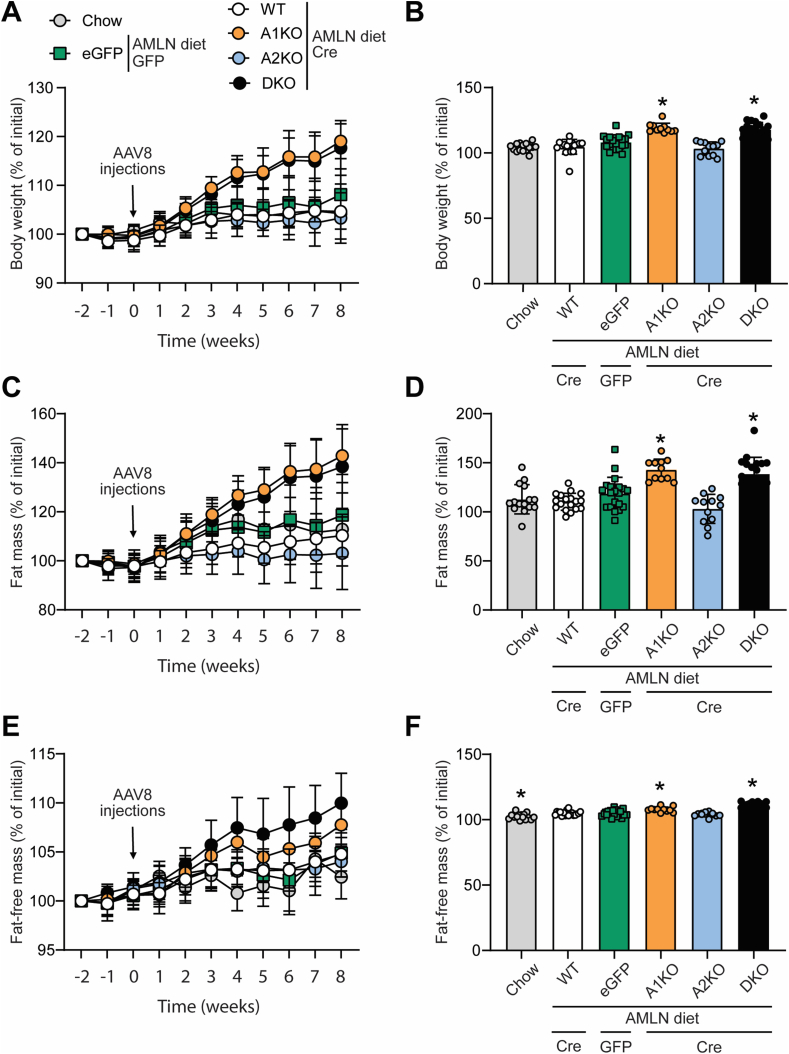
H**epatic loss of ACC1alone is sufficient to increase body mass in mice fed Amylin diet.** (A,B) Body weight measurements represented (A) as percentage of baseline over time and (B) at the end of the study. (C,D) Fat mass measurements represented (C) as percentage of baseline over time and (D) at the end of the study. (E,F) Lean mass measurements represented (E) as percentage of baseline over time and (F) at the end of the study. ∗indicates p < 0.05 compared to WT, determined by (B) Kruskal–Wallis test and (D,F) one-way ANOVA followed by Dunn's and Dunnett's multiple comparison post hoc test, respectively. Values are represented as mean ± SD, n = 11–20. Abbreviation: AMLN, Amylin diet.

Weekly body weight measurements showed that A1KO and DKO mice significantly increased their body mass over the course of the study (Figure 4A), resulting in a ∼18% body weight gain at the end of the study compared to WT controls (Fig. 4B). Body composition measures revealed that the weight gain in A1KO and DKO mice resulted from increased fat mass (29% and 26% greater than WT control, respectively, Figure 4C,D) and lean mass (3% and 5% greater than WT control, respectively, Figure 4E,F). In contrast, A2KO mice did not have a body composition phenotype different from WT, eGFP, or chow controls (Figure 4A–F).

**Figure 5:**
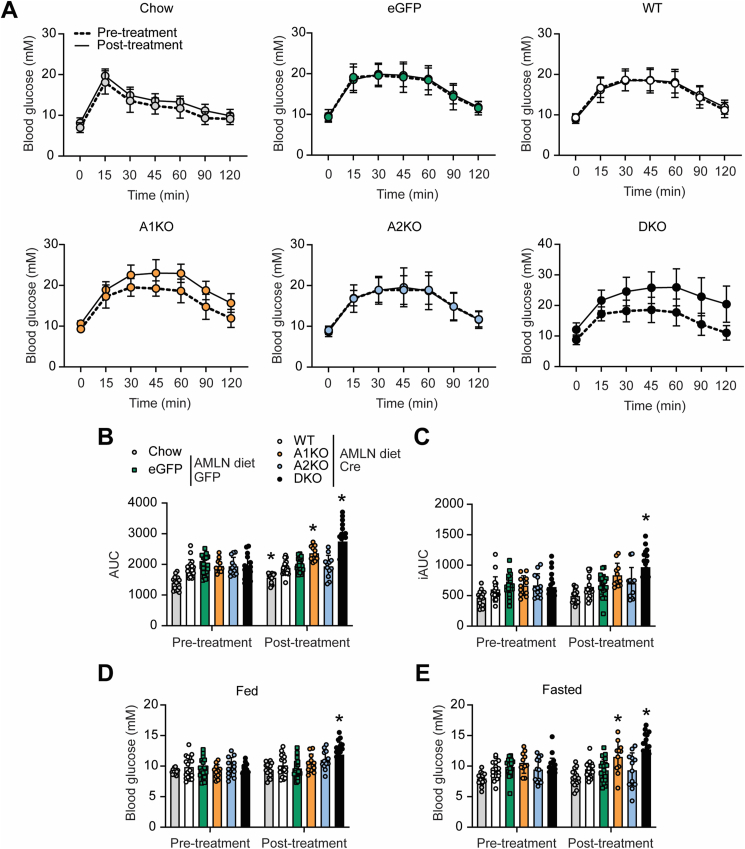
**Hepatic loss of ACC1 is sufficient to promote glucose intolerance in mice fed Amylin diet.** (A) Glucose tolerance tests (GTT) performed pre- and post-AAV8 injections (pre-treatment at week −2 and post-treatment at week 6). (B) Total area under the curve (AUC) and (C) incremental AUC pre- and post-treatment. (D) Random-fed and (E) fasted blood glucose measurements pre- and post-treatment. ∗indicates p < 0.05 compared to WT, determined by two-way repeated measures ANOVA followed by Dunnett's multiple comparison post hoc test. Values are represented as mean ± SD, n = 11–20. Abbreviations: AMLN, Amylin diet; iAUC, incremental AUC.

To evaluate the effects of selective and liver-targeted ACC inhibition on whole-body glucose homeostasis, we performed glucose tolerance tests (GTT) prior to AAV8 injections as well as two weeks before the end of the study (6 weeks post-treatment). The GTT data showed that A1KO and DKO mice had impaired glucose clearance six weeks after AAV8 injections (Figure 5A–C). DKO mice showed glucose intolerance by both total area under the curve (AUC) and incremental area under the curve (iAUC), while A1KO mice had significantly higher total AUC but not iAUC (Figure 5B,C). DKO mice had elevated blood glucose in both the fed and fasted states while the A1KO mice had elevated blood glucose in the fasted state only (Fig. 5E). In contrast, ACC2KO mice had normal glucose tolerance and blood glucose comparable to WT and eGFP controls (Figure 5A–E).

**Figure 6:**
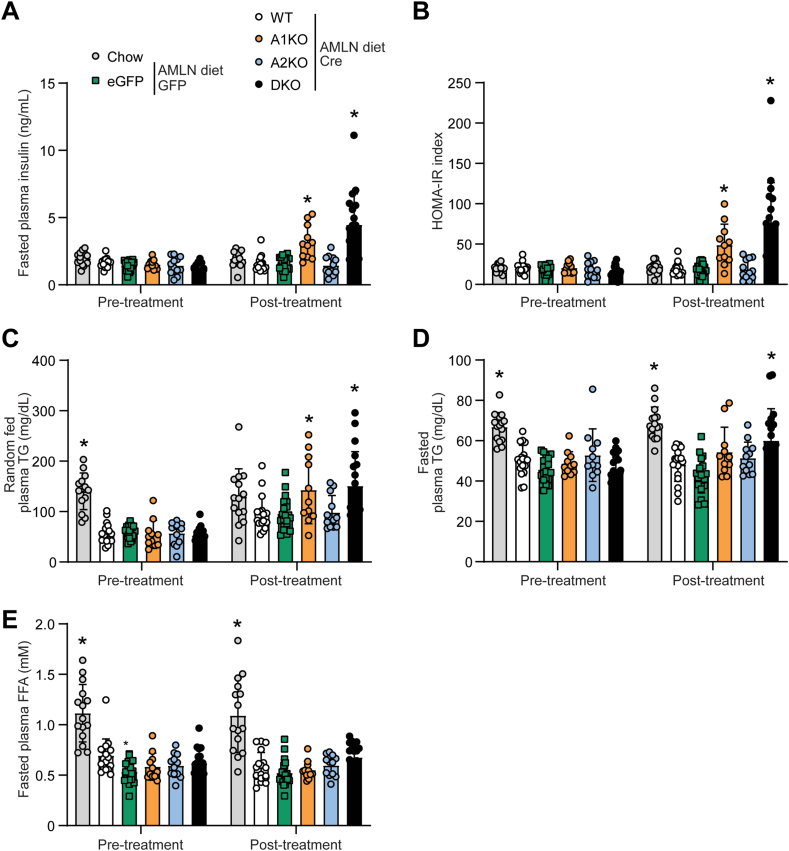
**Hepatic loss of ACC1 alone increases plasma insulin and triglyceride levels in mice fed Amylin diet.** (A) Fasted plasma insulin measurements and (B) calculated HOMA-IR index pre- and post-AAV8 injections (pre-treatment at week −2 and post-treatment at week 4). Plasma triglyceride (TG) levels in the random-fed (C) and fasted (D) states pre- and post-treatment. (E) Random-fed plasma free fatty acid (FFA) levels pre- and post-treatment. ∗indicates p < 0.05 compared to WT, determined by two-way repeated measures ANOVA followed by Dunnett's multiple comparison post hoc test. Values are represented as mean ± SD, n = 11–20. Abbreviation: AMLN, Amylin diet.

Insulin resistance and hyperlipidemia are frequently associated with MASH in mice and humans [2,34,35]; therefore, we measured plasma levels of insulin, triglyceride (TG) and free fatty acids (FFA) four weeks after AAV8 injections. We observed that A1KO and DKO mice were hyperinsulinemic (Figure 6A) and showed a significant increase in the HOMA-IR (Fig. 6B), which is an indicator of insulin resistance [29,30]. In addition, A1KO and DKO mice had elevated plasma TG levels that were 49% and 57% higher, respectively, than WT controls in the fed state (Fig. 6C) and DKO mice maintained elevated fasting TG levels that were 25% higher than WT controls (Fig. 6D). Fasted plasma FFA levels were unchanged by ACC isotype deletion pre-vs post-treatment (Fig. 6E). Mice lacking only liver ACC2 had similar plasma insulin, TG, and FFA levels comparable to WT controls (Figure 6A–E).

## 4 Discussion

ACC inhibitors are emerging as a promising therapeutic approach for a range of metabolic disorders including fatty liver disease [36,37]. However, both preclinical and clinical studies have shown that complete inhibition of ACC enzymes can lead to undesirable metabolic dysregulations, such as hypertriglyceridemia. It remains unclear whether the inhibition of either ACC1 or ACC2 alone systemically or liver-targeted represents a better strategy for reversing fatty liver without causing metabolic complications. In this study, we report the first preclinical investigation using a genetic approach for assessing the therapeutic potential of liver-specific inhibition of ACC1 and/or ACC2 in reversing MASH and liver fibrosis. To achieve hepatocyte-specific gene ablation, we utilized conditional mice with floxed ACC1 and/or ACC2 alleles and injected them with AAV-Cre virus with an AAV8 serotype (hepatocyte-selective) and a TBG promoter to achieve hepatocyte-targeted specificity. This experimental design closely mimics therapeutic interventions that would be applied in patients with established MASH, offering translational relevance beyond traditional germline knockout models. Quantitative PCR analyses revealed a 92–97% efficiency in ACC gene suppression, with residual transcript levels possibly reflecting contributions from non-hepatocyte liver cells.

We observed that mice lacking hepatocyte ACC1 or both ACC isotypes gained more weight over the course of the study compared to WT controls, with EchoMRI measures showing a ∼40% increase in adiposity compared to baseline. In addition, A1KO and DKO mice also developed glucose intolerance, while A2KO mice showed no changes compared to WT controls. These results were somewhat unexpected, as most previous studies in ACC1 or double ACC1/2 KO mice did not show effects on adiposity or glucose tolerance [24,28,38,39]. However, one recent study showed that liver-specific double ACC1/2 KO mice developed impaired glycemic regulation due to heightened gluconeogenesis [40], but adiposity was not assessed and the diet was high fat and high carbohydrate but not nutritionally comparable to the Amylin diet used in our study (which contains additional cholesterol, fructose, and trans-fat). The most closely-related phenotype to our study showed that the liver-targeted dual ACC inhibitor firsocostat increased adiposity and hyperglycemia in mice fed a MASH diet D09100301 (Research Diets) that has a very similar nutritional composition (40% kcal fat (mostly primex trans-fat), 2% cholesterol, and 20% kcal fructose) compared to our Amylin diet [41]. Therefore there is now complimentary pharmacological [41] and genetic (this study) evidence to support that loss of ACC1 activity impairs glucose tolerance in mice fed Amylin diet. However, limitations of our study include the use of an ip GTT instead of an oral GTT that may have resulted in greater glucose-stimulated insulin secretion, and the lack of insulin measurements during the GTT. Furthermore, the glucose dose was administered per unit lean mass; therefore, the modest relative increase of 3–5% lean mass in the ACC1 and DKO mice necessitated a mild increase in glucose dose compared to WT mice. The mechanism and biological significance of the mild increase in lean mass is unclear, but may be secondary to carrying 26–29% more adiposity relative to WT mice. The glucose tolerance test was a secondary readout to MASH pathophysiology and the glucose intolerance phenotype was not followed up or studied in detail. Therefore, future studies are needed to investigate the mechanisms linking ACC1 inhibition to glucose tolerance in mice fed Amylin diet using more sophisticated techniques including hyperinsulinemic-euglycemic clamp.

Further investigation is also required to elucidate the molecular mechanisms leading to increased adiposity and hyperglycemia upon increased fructose feeding in the absence of hepatic ACC1. Evidence from both human and animal studies shows that fructose is a potent inducer of hepatic DNL [42] and hepatocellular carcinoma (HCC) [43]. In these studies, the authors show that increased DNL not only contributes to ectopic fat deposition (i.e. in the liver), but it also impairs several metabolic processes through DNL-related fatty acids (e.g. beta-cell function, insulin secretion, or insulin sensitivity). Importantly, it has been reported that fructose intake promotes liver metabolic dysregulations more effectively than glucose via upregulation of lipogenic enzymes [44,45], even in healthy individuals [46]. We can therefore speculate that chronic exposure to high fructose diets may trigger enhanced metabolic dysregulations in the absence of hepatic ACC1, possibly due to compensatory mechanisms involving upregulation of other lipogenic enzymes such as those regulated by SREBP-1c, as previously reported by Kim et al. [20]. Considering that the consumption of sugar-sweetened beverages has increased dramatically in the last decades and that most of commercially available beverages in the United States are enriched with high-fructose corn syrup [15], our findings are clinically relevant in view of the current development of dual ACC-targeting drugs for treating MASLD.

On the other hand, in the present study we show that hepatocyte deletion of ACC2 in adult mice had negligible effects on mouse adiposity, glucose homeostasis and hepatic steatosis. Abu-Elheiga et al. previously reported that whole-body deletion of ACC2 from the embryonic stage prevented hepatic steatosis and insulin resistance under HFHC dietary feeding [47]. Previous studies from the same group [[48], [49], [50]] also showed a lean phenotype with improved insulin action due to increased whole-body, muscle fatty acid oxidation and higher energy expenditure, supporting the hypothesis that enhancing fatty acid utilization by deleting ACC2 leads to increased fatty acid utilisation and reduced fat stores. Similarly, Takagi et al. showed that ACC2 KO mice were protected from HFHC-induced weight gain and insulin resistance [51]. However, these works differ from our findings in a separate line of ACC2 KO mice [25], showing that whole-body deletion of ACC2 had no effect on adiposity or glucose tolerance, both under chow/HFHC feeding and under standard/thermoneutrality conditions [25,52]. Olson et al. have also separately observed that deletion of ACC2 had little effect on body weight and fat mass, both in whole-body and in muscle-specific KO mouse models [53]. Possible reasons for the phenotypic discrepancies in ACC2 deficient mice include different mouse genetic backgrounds, differences in diet composition and diet duration, distinct cloning strategies for generating mouse lines including retention of gene selection cassettes in the knockout but not control [54], and ACC gene deletion at the embryonic, neonatal, or adult stage. These discrepancies, together with existing evidence supporting significant functional overlap between ACC isotypes [55,56], suggest that the relative importance of each ACC isotype may depend on tissue-specific expression, age at ACC gene deletion, and diet/metabolic context. It is important to note that the studies cited herein are all developmental models where ACC genes are deleted early in life where ACC loss may be subject to compensation, which contrasts to the current study where ACC genes are deleted in fully developed adult mice.

We found that liver-targeted dual ACC1 and ACC2 inhibition in adult mice robustly improves liver MASH phenotypes but significantly increases plasma TG. These results are in line with previous findings using liver-targeted dual ACC1 and ACC2 inhibitors MK-4074 [20], Compound 1 [23] and firsocostat [57,58]. Our study is the first genetic KO study using a MASH reversal strategy where ACC genes are deleted after MASH onset, as opposed to a prevention strategy where ACC genes are deleted early in life. We also reveal the major impact that hepatic ACC1 has in modulating plasma TG as opposed to ACC2, as we observed that A1KO mice developed hypertriglyceridemia over the course of the study while A2KO mice did not. Tamura et al. previously tested Compound-1, an ACC1-selective inhibitor that is not liver-specific, in a MASH mouse model, and found that it remarkably decreased hepatic steatosis and fibrosis but retained the adverse effect of hypertriglyceridemia [59]. Our work has thus identified the liver as the key tissue orchestrating the ACC1-dependent regulation of plasma TG. As originally described for MK-4074 [20], but also observed with PF-05221304 [21], the increase in circulating triglycerides is likely mediated by the effects of decreased liver polyunsaturated fatty acids (PUFA) to stimulate SREBP1c activation, leading to increased glycerol-3-phosphate acyltransferase and very-low-density lipoprotein (VLDL) secretion. Collectively, the evidence to date suggests that ACC1 inhibition promotes VLDL export from the liver to prevent steatosis but consequently results in hypertriglyceridemia. However, a limitation of this study is that we did not assess hepatic VLDL secretion and therefore future studies are needed to determine the mechanism linking dual ACC1 and ACC2 inhibition to fasting hypertriglyceridemia.

In the present study, we demonstrated that neither ACC1 nor ACC2 hepatic inhibition alone is sufficient to reverse MASH, supporting a functional overlap between hepatic ACC isotypes while also indicating that hepatic loss of both isotypes is required to achieve a significant resolution of fatty liver steatohepatitis and liver fibrosis. Remarkably, we observed that liver-targeted ablation of ACC1 and ACC2 for a period of 8 weeks resulted in a ∼75% decrease of liver TG compared to WT controls, which is ∼2.5 fold larger than the decrease observed by Gapp et al. when mice were treated for 12 weeks with firsocostat under similar dietary feeding [41]. Furthermore, in our study DKO mice showed a marked regression of liver fibrosis stage, along with a robust gene expression suppression of all fibrosis markers tested, while ACC1 or ACC2 deletion alone had little effect on liver fibrosis markers. This finding has an important clinical relevance, as fibrosis regression is a key therapeutic goal for MASH therapies. While the driving factors are yet to be fully elucidated, liver fibrosis is largely driven by the crosstalk of steatotic hepatocytes with macrophages and hepatic stellate cells. Previous studies showed that firsocostat directly suppressed the activation of hepatic stellate cells, which are the primary cell type responsible for generating fibrotic scar tissue in the liver [60]. However, it is relevant to point out that firsocostat did not achieve improvement of MASH without worsening liver fibrosis in a Phase 2 clinical trial [57]. Firsocostat is a substrate for the hepatic organic anion-transporting polypeptide transporter, which thereby promotes higher hepatic concentrations [61], it is reasonable to hypothesise that better hepatocyte-specific targeting may be necessary to improve the efficacy of other ACC1/2 inhibitors to reverse fibrosis.

To the best of our knowledge, firsocostat and clesacostat are the only ACC inhibitors that are currently being tested in clinical trials for MASH [14], and they are both dual inhibitors and liver-targeted drugs; however, to avoid the hypertriglyceridemia side effect, they are being co-administered with ervogastat (NCT03248882) or cilofexor +/− semaglutide (NCT04971785) in Phase 2b trials. So far, multi-drug administration is showing a promising effect to resolve the adverse outcome of ACC inhibitors; however, monotherapies would generally be more desirable for easier administration, greater compliance, and avoidance of possible drug–drug interactions. The results from our study suggest that the complete inhibition of ACC2 with partial inhibition of ACC1 may represent a possible approach to reverse fatty liver disease phenotypes without hypertriglyceridemia.

In support of ACC2-selective targeting, recent evidence supports the development of ACC2 inhibitors as an effective approach for treating metabolic diseases. For example, TLC-3595, from OrsoBio, Inc., is a selective ACC2 inhibitor showing promising results for treating type 2 diabetes by increasing fatty acid oxidation, reducing ectopic lipid accumulation, and improving insulin sensitivity in skeletal muscle and liver of Zucker Diabetic Fatty (ZDF) rats and *db/db* mice [62,63]. OrsoBio, Inc. is currently running a Phase 2 clinical trial with this compound as a novel type 2 diabetes therapy (NCT05665751), but this compound may have potential as a treatment for MASH and related complications. In a previous study from our group we observed that ACC2-selective inhibition while maintaining total or partial activity at ACC1 trended to have the best anti-cancer phenotypes in a diethylnitrosamine-induced mouse model of hepatocellular carcinoma, resulting in a 50% decrease in tumour multiplicity as compared to controls [28]. Additionally, in the present study we found a significant decrease in mRNA levels of lysyl oxidase-like homolog 2 (LOXL2) in both A2KO and DKO livers. This protein is emerging as one of the major mediators of HCC progression and staging [64] and it is overexpressed in human HCC tissue compared to healthy liver tissue [65] at both the mRNA and protein levels.

Additional limitations of this study include the use of only male mice and only one treatment duration. Additional studies with female mice would determine whether the phenotypes observed are sex-dependent, and longer duration of treatment may provide more time to observe greater efficacy, particularly for fibrosis. Another limitation of this study is that we did not assess the metabolic phenotype of each genotype in chow-fed mice, which would have enabled a better understanding of the role of Amylin diet in the observed adverse phenotypes including insulin resistance and excess fat gain. Collectively, the results of this study identify that complete inhibition of both ACC1 and ACC2 resulted in robust reversal of MASH and fibrosis, while the deleterious phenotypes were largely driven by loss of ACC1.

Future work is needed to determine whether partial ACC1 inhibition with complete ACC2 inhibition may represent a better therapeutic strategy for reversing MASH and liver fibrosis while minimizing unwanted metabolic phenotypes of hypertriglyceridemia and glucose intolerance. A similar liver-targeted ACC1-sparing approach with complete inhibition of ACC2 conferred the best resistance to carcinogen-induced liver cancer in mice [28]. Therefore, an ACC inhibitor that completely inhibits liver ACC2 while sparing some ACC1 activity may represent a potentially refined approach to treat MASH and liver fibrosis.

## CRediT authorship contribution statement

**Martina Beretta:** Writing – review & editing, Writing – original draft, Methodology, Investigation, Formal analysis, Data curation, Conceptualization. **Calum S. Vancuylenburg:** Writing – review & editing, Investigation. **Riya Shrestha:** Writing – review & editing, Methodology, Investigation. **Ellen M. Olzomer:** Writing – review & editing, Investigation. **Brenna Osborne:** Investigation, Writing – review & editing. **Mingyan Zhou:** Investigation, Writing – review & editing. **Suri Zhang:** Investigation, Writing – review & editing. **Adam Hargreaves:** Writing – review & editing, Investigation. **Frances L. Byrne:** Writing – review & editing, Validation, Supervision, Resources. **Kyle L. Hoehn:** Writing – review & editing, Writing – original draft, Validation, Supervision, Resources, Project administration, Methodology, Funding acquisition, Conceptualization.

## Disclosures

K.L.H. declares commercial interest in Life Biosciences and Uncoupler Biosciences. All other authors have no conflicts of interest.

## Funding

This work was supported by NHMRC grants GNT1163903 and GNT2014079. R.S. is supported by a UNSW Scientia PhD Scholarship.

## Declaration of competing interest

The authors declare the following financial interests/personal relationships which may be considered as potential competing interests: **K.L.H.** declares commercial interest in Life Biosciences and Uncoupler Biosciences that is unrelated to ACC inhibitor therapies. **All other authors** declare no competing financial interests or personal relationships that could have appeared to influence the work reported in this paper.

## Data Availability

Data will be made available on request.

## References

[1] Le P., Tatar M., Dasarathy S., Alkhouri N., Herman W.H., Taksler G.B. (2025). Estimated burden of metabolic dysfunction-associated steatotic liver disease in US adults, 2020 to 2050. JAMA Netw Open.

[2] Younossi Z.M., Golabi P., Paik J.M., Henry A., Van Dongen C., Henry L. (2023). The global epidemiology of nonalcoholic fatty liver disease (NAFLD) and nonalcoholic steatohepatitis (NASH): a systematic review. Hepatology.

[3] Lee E.J., Choi M., Ahn S.B., Yoo J.J., Kang S.H., Cho Y. (2024). Prevalence of nonalcoholic fatty liver disease in pediatrics and adolescents: a systematic review and meta-analysis. World J Pediatr.

[4] Diehl A.M., Day C. (2017). Cause, pathogenesis, and treatment of nonalcoholic steatohepatitis. N Engl J Med.

[5] Dulai P.S., Singh S., Patel J., Soni M., Prokop L.J., Younossi Z. (2017). Increased risk of mortality by fibrosis stage in nonalcoholic fatty liver disease: systematic review and meta-analysis. Hepatology.

[6] Hagstrom H., Nasr P., Ekstedt M., Hammar U., Stal P., Hultcrantz R. (2017). Fibrosis stage but not NASH predicts mortality and time to development of severe liver disease in biopsy-proven NAFLD. J Hepatol.

[7] Sanyal A.J., Van Natta M.L., Clark J., Neuschwander-Tetri B.A., Diehl A., Dasarathy S. (2021). Prospective study of outcomes in adults with nonalcoholic fatty liver disease. N Engl J Med.

[8] Vilar-Gomez E., Martinez-Perez Y., Calzadilla-Bertot L., Torres-Gonzalez A., Gra-Oramas B., Gonzalez-Fabian L. (2015). Weight loss through lifestyle modification significantly reduces features of nonalcoholic steatohepatitis. Gastroenterology.

[9] Promrat K., Kleiner D.E., Niemeier H.M., Jackvony E., Kearns M., Wands J.R. (2010). Randomized controlled trial testing the effects of weight loss on nonalcoholic steatohepatitis. Hepatology.

[10] Alshehade S.A. (2024). Resmetirom's approval: highlighting the need for comprehensive approaches in NASH therapeutics. Clin Res Hepatol Gastroenterol.

[11] Harrison S.A., Bedossa P., Guy C.D., Schattenberg J.M., Loomba R., Taub R. (2024). A phase 3, randomized, controlled trial of resmetirom in NASH with liver fibrosis. N Engl J Med.

[12] Lambert J.E., Ramos-Roman M.A., Browning J.D., Parks E.J. (2014). Increased de novo lipogenesis is a distinct characteristic of individuals with nonalcoholic fatty liver disease. Gastroenterology.

[13] Smith G.I., Shankaran M., Yoshino M., Schweitzer G.G., Chondronikola M., Beals J.W. (2020). Insulin resistance drives hepatic de novo lipogenesis in nonalcoholic fatty liver disease. J Clin Investig.

[14] Esler W.P., Cohen D.E. (2024). Pharmacologic inhibition of lipogenesis for the treatment of NAFLD. J Hepatol.

[15] Mateo-Marin M.A., Alves-Bezerra M. (2024). Targeting acetyl-CoA carboxylases for the treatment of MASLD. J Lipid Res.

[16] Wang Y., Yu W., Li S., Guo D., He J., Wang Y. (2022). Acetyl-CoA carboxylases and diseases. Front Oncol.

[17] Harada N., Oda Z., Hara Y., Fujinami K., Okawa M., Ohbuchi K. (2007). Hepatic de novo lipogenesis is present in liver-specific ACC1-deficient mice. Mol Cell Biol.

[18] Bian H., Liu Y.M., Chen Z.N. (2022). New avenues for NASH therapy by targeting ACC. Cell Metab.

[19] Kelly K.L., Reagan W.J., Sonnenberg G.E., Clasquin M., Hales K., Asano S. (2020). De novo lipogenesis is essential for platelet production in humans. Nat Metab.

[20] Kim C.W., Addy C., Kusunoki J., Anderson N.N., Deja S., Fu X. (2017). Acetyl CoA carboxylase inhibition reduces hepatic steatosis but elevates plasma triglycerides in mice and humans: a bedside to bench investigation. Cell Metab.

[21] Calle R.A., Amin N.B., Carvajal-Gonzalez S., Ross T.T., Bergman A., Aggarwal S. (2021). ACC inhibitor alone or co-administered with a DGAT2 inhibitor in patients with non-alcoholic fatty liver disease: two parallel, placebo-controlled, randomized phase 2a trials. Nat Med.

[22] Loomba R., Kayali Z., Noureddin M., Ruane P., Lawitz E.J., Bennett M. (2018). GS-0976 reduces hepatic steatosis and fibrosis markers in patients with nonalcoholic fatty liver disease. Gastroenterology.

[23] Goedeke L., Bates J., Vatner D.F., Perry R.J., Wang T., Ramirez R. (2018). Acetyl-CoA carboxylase inhibition reverses NAFLD and hepatic insulin resistance but promotes hypertriglyceridemia in rodents. Hepatology.

[24] Chow J.D., Lawrence R.T., Healy M.E., Dominy J.E., Liao J.A., Breen D.S. (2014). Genetic inhibition of hepatic acetyl-CoA carboxylase activity increases liver fat and alters global protein acetylation. Mol Metab.

[25] Hoehn K.L., Turner N., Swarbrick M.M., Wilks D., Preston E., Phua Y. (2010). Acute or chronic upregulation of mitochondrial fatty acid oxidation has no net effect on whole-body energy expenditure or adiposity. Cell Metab.

[26] Kleiner D.E., Brunt E.M., Van Natta M., Behling C., Contos M.J., Cummings O.W. (2005). Design and validation of a histological scoring system for nonalcoholic fatty liver disease. Hepatology.

[27] Folch J., Lees M., Sloane Stanley G.H. (1957). A simple method for the isolation and purification of total lipides from animal tissues. J Biol Chem.

[28] Shrestha R., Vancuylenburg C.S., Beretta M., Zhou M., Shah D.P., Olzomer E.M. (2024). Complete inhibition of liver acetyl-CoA carboxylase activity is required to exacerbate liver tumorigenesis in mice treated with diethylnitrosamine. Cancer Metabol.

[29] Cacho J., Sevillano J., de Castro J., Herrera E., Ramos M.P. (2008). Validation of simple indexes to assess insulin sensitivity during pregnancy in Wistar and sprague-dawley rats. Am J Physiol Endocrinol Metab.

[30] Berglund E.D., Li C.Y., Poffenberger G., Ayala J.E., Fueger P.T., Willis S.E. (2008). Glucose metabolism in vivo in four commonly used inbred mouse strains. Diabetes.

[31] Clapper J.R., Hendricks M.D., Gu G., Wittmer C., Dolman C.S., Herich J. (2013). Diet-induced mouse model of fatty liver disease and nonalcoholic steatohepatitis reflecting clinical disease progression and methods of assessment. Am J Physiol Gastrointest Liver Physiol.

[32] Trevaskis J.L., Griffin P.S., Wittmer C., Neuschwander-Tetri B.A., Brunt E.M., Dolman C.S. (2012). Glucagon-like peptide-1 receptor agonism improves metabolic, biochemical, and histopathological indices of nonalcoholic steatohepatitis in mice. Am J Physiol Gastrointest Liver Physiol.

[33] Tolbol K.S., Kristiansen M.N., Hansen H.H., Veidal S.S., Rigbolt K.T., Gillum M.P. (2018). Metabolic and hepatic effects of liraglutide, obeticholic acid and elafibranor in diet-induced obese mouse models of biopsy-confirmed nonalcoholic steatohepatitis. World J Gastroenterol.

[34] Scoditti E., Sabatini S., Carli F., Gastaldelli A. (2024). Hepatic glucose metabolism in the steatotic liver. Nat Rev Gastroenterol Hepatol.

[35] Carli F., Della Pepa G., Sabatini S., Vidal Puig A., Gastaldelli A. (2024). Lipid metabolism in MASLD and MASH: from mechanism to the clinic. JHEP Rep.

[36] Batchuluun B., Pinkosky S.L., Steinberg G.R. (2022). Lipogenesis inhibitors: therapeutic opportunities and challenges. Nat Rev Drug Discov.

[37] Imai N., Cohen D.E. (2018). Trimming the fat: acetyl-coa carboxylase inhibition for the management of NAFLD. Hepatology.

[38] Nelson M.E., Lahiri S., Chow J.D., Byrne F.L., Hargett S.R., Breen D.S. (2017). Inhibition of hepatic lipogenesis enhances liver tumorigenesis by increasing antioxidant defence and promoting cell survival. Nat Commun.

[39] Mao J., DeMayo F.J., Li H., Abu-Elheiga L., Gu Z., Shaikenov T.E. (2006). Liver-specific deletion of acetyl-CoA carboxylase 1 reduces hepatic triglyceride accumulation without affecting glucose homeostasis. Proc Natl Acad Sci U S A.

[40] Deja S., Fletcher J.A., Kim C.W., Kucejova B., Fu X., Mizerska M. (2024). Hepatic malonyl-CoA synthesis restrains gluconeogenesis by suppressing fat oxidation, pyruvate carboxylation, and amino acid availability. Cell Metab.

[41] Gapp B., Jourdain M., Bringer P., Kueng B., Weber D., Osmont A. (2020). Farnesoid X Receptor Agonism, Acetyl-Coenzyme A Carboxylase Inhibition, and Back Translation of Clinically Observed Endpoints of De Novo Lipogenesis in a Murine NASH Model. Hepatol Commun.

[42] Geidl-Flueck B., Gerber P.A. (2023). Fructose drives de novo lipogenesis affecting metabolic health. J Endocrinol.

[43] Hargett S., Lahiri S., Kowalski G.M., Corley S., Nelson M.E., Lackner C. (2024). Bile acids mediate fructose-associated liver tumour growth in mice. Biochim Biophys Acta Mol Basis Dis.

[44] Janevski M., Ratnayake S., Siljanovski S., McGlynn M.A., Cameron-Smith D., Lewandowski P. (2012). Fructose containing sugars modulate mRNA of lipogenic genes ACC and FAS and protein levels of transcription factors ChREBP and SREBP1c with no effect on body weight or liver fat. Food Funct.

[45] Kim M.S., Krawczyk S.A., Doridot L., Fowler A.J., Wang J.X., Trauger S.A. (2016). ChREBP regulates fructose-induced glucose production independently of insulin signaling. J Clin Investig.

[46] Geidl-Flueck B., Hochuli M., Nemeth A., Eberl A., Derron N., Kofeler H.C. (2021). Fructose- and sucrose- but not glucose-sweetened beverages promote hepatic de novo lipogenesis: a randomized controlled trial. J Hepatol.

[47] Abu-Elheiga L., Wu H., Gu Z., Bressler R., Wakil S.J. (2012). Acetyl-CoA carboxylase 2-/- mutant mice are protected against fatty liver under high-fat, high-carbohydrate dietary and de novo lipogenic conditions. J Biol Chem.

[48] Abu-Elheiga L., Oh W., Kordari P., Wakil S.J. (2003). Acetyl-CoA carboxylase 2 mutant mice are protected against obesity and diabetes induced by high-fat/high-carbohydrate diets. Proc Natl Acad Sci U S A.

[49] Choi C.S., Savage D.B., Abu-Elheiga L., Liu Z.X., Kim S., Kulkarni A. (2007). Continuous fat oxidation in acetyl-CoA carboxylase 2 knockout mice increases total energy expenditure, reduces fat mass, and improves insulin sensitivity. Proc Natl Acad Sci U S A.

[50] Abu-Elheiga L., Matzuk M.M., Abo-Hashema K.A., Wakil S.J. (2001). Continuous fatty acid oxidation and reduced fat storage in mice lacking acetyl-CoA carboxylase 2. Science.

[51] Takagi H., Ikehara T., Kashiwagi Y., Hashimoto K., Nanchi I., Shimazaki A. (2018). ACC2 deletion enhances IMCL reduction along with Acetyl-CoA metabolism and improves insulin sensitivity in Male mice. Endocrinology.

[52] Brandon A.E., Stuart E., Leslie S.J., Hoehn K.L., James D.E., Kraegen E.W. (2016). Minimal impact of age and housing temperature on the metabolic phenotype of Acc2-/- mice. J Endocrinol.

[53] Olson D.P., Pulinilkunnil T., Cline G.W., Shulman G.I., Lowell B.B. (2010). Gene knockout of Acc2 has little effect on body weight, fat mass, or food intake. Proc Natl Acad Sci U S A.

[54] Hoehn K.L., Turner N., Cooney G.J., James D.E. (2012). Phenotypic discrepancies in acetyl-CoA carboxylase 2-deficient mice. J Biol Chem.

[55] Savage D.B., Choi C.S., Samuel V.T., Liu Z.X., Zhang D., Wang A. (2006). Reversal of diet-induced hepatic steatosis and hepatic insulin resistance by antisense oligonucleotide inhibitors of acetyl-CoA carboxylases 1 and 2. J Clin Investig.

[56] Fullerton M.D., Galic S., Marcinko K., Sikkema S., Pulinilkunnil T., Chen Z.P. (2013). Single phosphorylation sites in Acc1 and Acc2 regulate lipid homeostasis and the insulin-sensitizing effects of metformin. Nat Med.

[57] Loomba R., Noureddin M., Kowdley K.V., Kohli A., Sheikh A., Neff G. (2021). Combination therapies including Cilofexor and firsocostat for bridging fibrosis and cirrhosis attributable to NASH. Hepatology.

[58] Devereux C.J., Bayliss J., Keenan S.N., Montgomery M.K., Watt M.J. (2023). Investigating dual inhibition of ACC and CD36 for the treatment of nonalcoholic fatty liver disease in mice. Am J Physiol Endocrinol Metab.

[59] Tamura Y.O., Sugama J., Iwasaki S., Sasaki M., Yasuno H., Aoyama K. (2021). Selective Acetyl-CoA carboxylase 1 inhibitor improves hepatic steatosis and hepatic fibrosis in a preclinical nonalcoholic steatohepatitis model. J Pharmacol Exp Ther.

[60] Bates J., Vijayakumar A., Ghoshal S., Marchand B., Yi S., Kornyeyev D. (2020). Acetyl-CoA carboxylase inhibition disrupts metabolic reprogramming during hepatic stellate cell activation. J Hepatol.

[61] Alkhouri N., Lawitz E., Noureddin M., DeFronzo R., Shulman G.I. (2020). GS-0976 (Firsocostat): an investigational liver-directed acetyl-CoA carboxylase (ACC) inhibitor for the treatment of non-alcoholic steatohepatitis (NASH). Expet Opin Invest Drugs.

[62] Vijayakumar A., Murakami E., Huss R.S., Sroda N., Shimazaki A., Kashiwagi Y. (2023). 849-P: antidiabetic effects of TLC-3595, a selective ACC2 inhibitor, in ZDF rats. Diabetes.

[63] Vijayakumar A., Sroda N., Murakami E., Weng S., Myers R.P., Subramanian M. (2024). 899-P: combinations of the mitochondrial protonophore TLC-6740 and/or the ACC2 inhibitor TLC-3595 provide additive glycemic benefits to semaglutide (SEMA) in db/db mice. Diabetes.

[64] Wong C.C., Tse A.P., Huang Y.P., Zhu Y.T., Chiu D.K., Lai R.K. (2014). Lysyl oxidase-like 2 is critical to tumor microenvironment and metastatic niche formation in hepatocellular carcinoma. Hepatology.

[65] Radic J., Kozik B., Nikolic I., Kolarov-Bjelobrk I., Vasiljevic T., Vranjkovic B. (2023). Multiple roles of LOXL2 in the progression of hepatocellular carcinoma and its potential for therapeutic targeting. Int J Mol Sci.

