# Supplementary material for: Isotype-selective roles of hepatic acetyl-CoA carboxylases in a mouse model of fatty liver disease
Source: Mol Metab. 2025 Oct 4;102:102264. doi: 10.1016/j.molmet.2025.102264 (PMC12555813; doi:10.1016/j.molmet.2025.102264)
Supplement: Multimedia component 1 [file mmc1.docx]

Supplementary Material

**Isotype-selective roles of hepatic acetyl-CoA carboxylases in a mouse model of fatty liver disease**

Martina Beretta^1^, Calum S Vancuylenburg^1^, Riya Shrestha^1^, Ellen M Olzomer^1^, Brenna Osborne^1^, Mingyan Zhou^1^, Suri Zhang^1^, Adam Hargreaves^2^, Frances L Byrne^1^, and Kyle L Hoehn^1*^

**S1. Supplementary Figures**

**
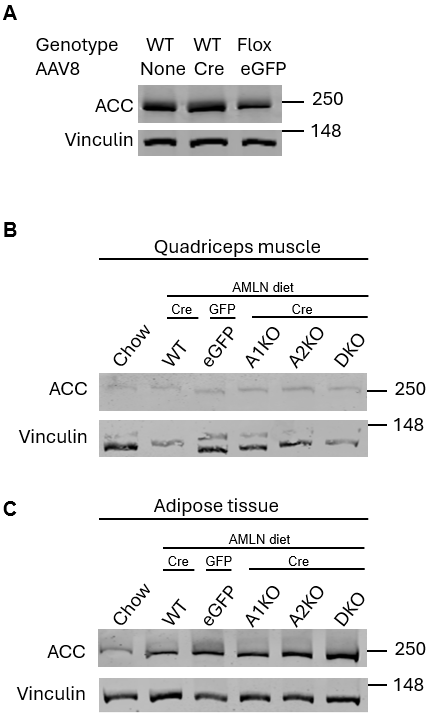
**

**Supplementary Figure S1. AAV8 treatment does not alter ACC enzyme expression and is liver-selective.** (A) Representative western blot comparing ACC expression in liver tissue from mice fed Amylin diet for ~7 months and treatment with no AAV8 virus, AAV8-TBG-Cre, or AAV8-TBG-eGFP as indicated. Representative western blot of ACC protein expression from (B) quadriceps muscle and (C) gonadal adipose tissue from each group at the study endpoint.

**
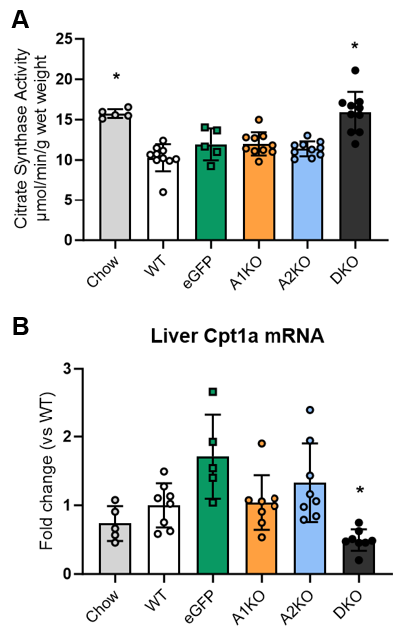
**

**Supplementary Figure S2. Validation of functional changes known to be altered by liver ACC1/ACC2 double knockout.** (A) Citrate synthase (CS) activity and (B) carnitine palmitoyltransferase 1a (*Cpt1a*) gene expression were evaluated in liver tissue from mice at the end of the study. Mice lacking both ACC isotypes in the liver had elevated CS activity and lower *Cpt1a* expression compared to WT control mice. In contrast, single isotype ACC1 or ACC2 deletion had no effect on CS activity or *Cpt1a* expression. (A) Average of duplicate runs with WT using 1-way ANOVA with Dunnett's correction for multiple comparisons. (B) Statistical significance determined by Kruskal-Wallis test followed by Dunn’s multiple comparison post hoc test. *indicates p<0.05 compared to WT. Values are represented as mean ± SD, n=5-10.

**
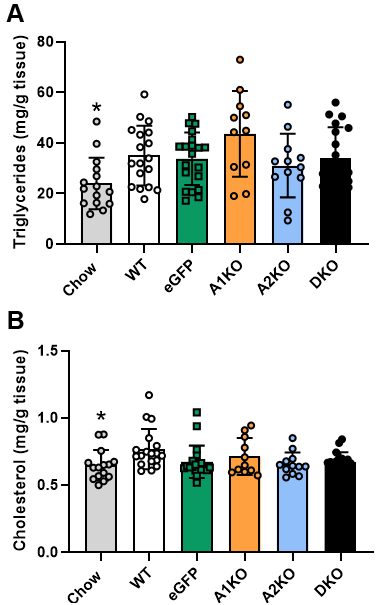
**

**Supplementary Figure S3. Liver ACC1 and ACC2 deletion does not impact muscle lipids in mice fed Amylin diet.** Quadriceps triglyceride (A) and cholesterol (B) levels measured from tissues collected at the end of the study. *indicates p<0.05 compared to WT, determined by Kruskal-Wallis test followed by Dunn’s multiple comparison post hoc test. Values are represented as mean ± SD, n=11-20.

**S2. Supplementary Methods**

**S2.1 Citrate Synthase activity**

Frozen liver tissue was manually homogenized 1:19 (wt/vol) in 50 mM Tris-HCl, 1 mM EDTA and 0.1% Triton X-100, pH 7.2 using a Polytron homogeniser (Kinetica, Sweden)). Liver homogenates were subjected to three freeze-thaw cycles and then centrifuged at 7,000 g for 10 minutes at 4°C. Supernatants were used to determine the activity of citrate synthase (CS) at 30°C on a temperature-controlled plate reader (EnSight, Perkin Elmer, CT, USA) as described previously (Liu et al). Liver homogenates were diluted 1:6 for the citrate synthase assay.  The reaction mixture was 100 mM Tris-HCl, 1 mM MgCl_2_, 1 mM EDTA, 1 mM [DTNB](https://www.sciencedirect.com/topics/medicine-and-dentistry/dtnb) (Ellman's Reagent), 0.4 mM acetyl-CoA, pH 8.2. 10 μl of 1/120 homogenate and 240 μl of reaction buffer was added to each well. The reaction was initiated with 50 μl of 6 mM [oxaloacetate](https://www.sciencedirect.com/topics/medicine-and-dentistry/oxaloacetic-acid). The reaction was followed at 412 nm for 5 min. Enzyme activities are presented as units per gram of wet weight, where units are defined as micromoles per minute.

**S2.2 Lipid and biochemical analysis**

Muscle lipids were analysed as described in the main manuscript text with the exception that quadriceps tissues were used instead of liver.

**S2.3 Quantitative real-time PCR (qRT-PCR)**

RNA extraction and qRT-PCR was performed as described in the main text. The primers for *Cpt1a* included forward primer ttgggccggttgctgat and reverse primer gtctcagggctagagaacttggaa that resulted in a 100 bp amplicon.
